# Conceptual model of managing health care volunteers in disasters: a mixed method study

**DOI:** 10.1186/s12913-019-4073-6

**Published:** 2019-04-24

**Authors:** Ibrahim Salmani, Hesam Seyedin, Ali Ardalan, Tahmineh Farajkhoda

**Affiliations:** 1 Department of Disasters and Emergency Health, Research Center of Accidents Prevention and Dealing with Disasters, Shahid Sadoughi University of Medical Sciences, Yazd, Iran; 2 Department of Health in Emergencies and Disasters, School of Health Management and Information Sciences, Iran University of Medical Sciences, Tehran, Iran; 3 Department of Disaster Public Health, School of Public Health, Tehran University of Medical Sciences, Tehran, Iran; 4 Harvard Humanitarian Initiative, Harvard University, Cambridge, USA; 5 Reproductive Health, Research Center for Nursing and Midwifery Care, Shahid Sadoughi University of Medical Sciences, Yazd, Iran

**Keywords:** Health staff, Volunteer, Volunteer management, Personnel management, Personnel administration, Hospital, Delivery of health care, Disaster planning, Disaster, Qualitative research

## Abstract

**Background:**

Shortage of specialized healthcare volunteers is a major challenge during disasters and one solution could be pre-identified healthcare volunteers. This study aimed to develop a conceptual model of managing Iranian healthcare volunteers in disasters.

**Methods:**

This mixed method study was designed in two phases. A qualitative study using semi-structured interviews was conducted with 22 health professionals and key informant. The interviews were analyzed by framework analysis. In the second phase, concepts derived from the first step were evaluated in a two round Delphi study by an expert panel comprised of 42 experts.

**Results:**

Two themes and eight subthemes were identified based on the results of the first phase. The theme of background requirements included three sub-themes of laws and regulations, NGOs and socio-cultural factors. The second theme was called operational requirements which included six subthemes of preparedness, response, retention, relocation, terminating and follow-up. According to the results of the Delphi study, all of the concepts were confirmed.

**Conclusion:**

In addition to the need for supportive legal framework and building the culture of volunteering, it seems it is crucial to identify and prepare the health care volunteers in the preparedness phase and assign them appropriately in the response phase. Furthermore, the necessary measures should be prioritized to enhance volunteers’ retention rate and motivation. Plans should also be implemented for volunteers’ termination and volunteers’ physical and mental health follow up after their mission.

## Background

Health system according to the WHO refers to all organizations, people and actions in which its primary goal is to promote, restore or maintain health [1] and play a crucial role in responding to disasters. Disasters could adversely affect health systems through destruction of healthcare facilities and shortage of personnel. For example, in the 2003 earthquake of Algeria, 50% and in the Bam earthquake in Iran, all hospitals and health centers were collapsed [2].

Damages to the health facilities during disasters and shortage of health human resources in response to disasters, can lead to irreparable consequences and increase the rate of morbidity and mortality [3]. Therefore, health systems must well organize and use the capacity of its system before disasters to enable them to deliver the maximum services in response phase [4]. In this regard, one of the challenges of capacity building is inability to quickly mobilize human resources at the time of disasters [5] as well as impractical use of by-standers and uninvited volunteers in disasters [6]. In such situations, one source of human resources is the use of professional pre-identified volunteers [7].

Iran has a high level of exposure to multiple hazards such as floods, dust storms and severe earthquakes [8]. According to EM-DAT, 33190 deaths and more than 3 million affected occurred during 210 disasters from 2000 to 2018 in Iran [9]. Although, Iran has made considerable progress in response to disasters, there are still numerous challenges in disaster risk reduction that should be addressed [10]. In Iran, the National Disaster Management Organization (NDMO) is affiliated to the Ministry of Interiors and is responsible for the management and coordination of all activities related to responding to disasters. The NDMO has established 14 technical national taskforces and a related ministry or organization coordinates each taskforce. One of these taskforce is Health and Medical Care, which is coordinated by Ministry of Health and is responsible for providing all health services for the affected people [10].

One of the methods of enhancing the capacity and responsiveness of the health system in disasters, which is in line with the second priority of the Sendai Framework for Disaster Risk Reduction [13] is increasing the quantity and quality of human resources by using volunteers [14]. One of the big issues in this system is lack of plan for organizing and employing volunteers in the health sector at the time of disasters [11]. Lack of plan makes it less likely to use these volunteers when there is a shortage of official forces. In Iran, however, there are many volunteers in disaster situations, but there are no specific rules and regulations for recruiting, organizing and using their capacities within the framework of the health system [12].

It seems that in the case of volunteer surge and lack of plan to manage their capacity will lead to overcrowding, their inconsistent and unprofessional interventions and failure to comply with the rules, safety reduction and waste of resources and time. The International Federation of Red Cross and Red Crescent Societies (IFRC) has already published some guidelines in the field of volunteer security [15], organization of volunteers and dealing with uninvited volunteers [16], legislation and volunteerism [17]. Additionally in Abplanalp’s study which is sponsored by IFRC, a cycle of managing volunteers has been presented [18]. The components of this model include imagine, plan, recruit, screen, place, train, motivate, support, recognize, access and transition of the volunteers.

Studies indicated that individuals are willing to provide services to people affected by disasters and there is strong evidence that pre-identified volunteers can play a valuable role in achieving disaster management goals in all its phases from mitigation to recovery [19]. In this regard, some previous studies have provided models to manage volunteers (in general, rather than in disasters), and one of the most known and oldest is the Voluntary Resource Management Model presented by Boyce in the early 1970s, known as ISOTURE (its letters stand for identification, orientation, training, utilization, recognition, and evaluation) [20]. Brudney also proposed five steps to prepare the volunteer management plan. These five steps, including: identifying the reasons for the volunteers’ need, obtaining organizational staffs’ consent, designing an appropriate organizational structure, and appointing a competent leader for the general guidance of volunteers. Stepputat also referred to ten effective steps to successfully manage volunteers that included recruitment, application, interview and screening, orientation and training, placement, supervision and evaluation, recognition, retention, record keeping, advocacy and education [21].

Considering the increasing risk of disasters; critical role of health system in responding to disasters; few studies in the field of disaster volunteering in the world [22]; and also lack of a comprehensive study addressing all aspects of the healthcare volunteer management in disasters, this study aimed to develop a conceptual model for appropriate management and using capacity of healthcare volunteers in disasters in Iran.

## Method

An exploratory sequential mixed-method [23] study was conducted in two sequential phases in 2017. The first phase was carried out qualitatively. The study population in the first phase consisted of disaster managers and volunteers themselves. Theoretical sampling was conducted using purposive sampling, snowball sampling and key informants interview. The inclusion criteria included sufficient knowledge and experience in human resource management and/or having at least three years of work experience in disaster management and managing volunteers or being a volunteer in at least two events.

Verbal consent was obtained from all participants, and no honorarium was offered. One of the researchers (I.S, Ph.D. student, male) conducted the face-to-face semi-structured interview. Interviews were recorded using a recording device, and field notes were written when needed. The interview questions included management status of healthcare volunteers in Iran and strategies for comprehensive management of these volunteers. After each interview, the recorded interview text was transcribed in Microsoft Word, and primary data analysis was performed after importing the Word file into MaxQDA 10.

Interviews were conducted from January 2017 to May 2017 with mean duration of 56 min. Sampling continued to reach data saturation. 22 interviews and one repeat interview were performed. Three individuals refused to participate due to the lack of time and interest. Framework analysis was used as a data analysis method, including seven steps; transcription, familiarization with interview, coding, developing a working analytical framework, applying the framework, charting data, and finally interpreting [24]. Accordingly, after transcription of the interviews by one of the authors (I.S.), all of the authors carefully read the transcriptions to become familiar with the whole interviews. After coding the first three transcripts, and considering the existing literature, the objectives of the study, and the comments of the researchers; a working analytical framework was developed. This framework was used for data analysis. However, the initial analytical framework applied to the subsequent transcripts. At the final stage, the data were charted into a framework matrix, and interpretation was later carried out.

Prolonged engagement and persistent observation, peer checking, and searching for disconfirming evidence were among the measures taken by the researchers to ensure the rigor of the qualitative research. To ensure the accuracy of the findings, the researchers respected the principle of prolonged engagement with interviewees at all stages of data collection [25]. Meanwhile, the interviewees were selected from different specialties (person triangulation), and sampling was performed in various cities (place triangulation).

For member checking, one of the encoded texts was returned to several participants, and their feedback was applied. The data dependency was performed through peer checking (two experts in qualitative framework analysis outside the research group), and the research team checking (supervisors and counselor) [26]. In addition, to increase the confirm-ability and credibility of the results, the researcher elaborated the research details and recorded all the stages of data collection.

In the second phase, the Delphi method was used to reach consensus on the constructs, which extracted in the first phase by the panel of experts. The panel consists of 42 distinguished experts. The selection criteria were holding a key position in managing disasters, or having professional experience or knowledge in the field of disasters and volunteer management in public and private institutions. The Delphi planned in three rounds [27]. In the first round, a questionnaire was prepared based on the items obtained from the first phase and the panel of experts specified the importance of each items in a five point Likert scale. Each Likert item was given a range of scores from one to five (completely disagree to completely agree). Then the percentage of total score of each question was calculated as consensus rate. Experts were invited to add any probable additional items in an open-ended question at the end of each item. The acceptable consensus rate was 75% and items with at least 75% of consensus were accepted. Items with consensus of 25 to 74% were remained in the next round and the items with less than 25% were eliminated. In the second round, the items with 25 to 74% of consensus (based on the results of the first round) were entered into the questionnaire. In this round, the participants were asked to specify the importance of each item in a five point Likert scales again.

In the third round, the experts were asked to specify their final opinion about the remaining items. Descriptive statistical tests including percentage and frequency were used to analyze the data of this phase.

## Results

### First phase

Out of the 22 participants in the first step, 86% were men and the mean age of the participants was 45 ± 6.5 years with 17 years’ relevant work experience.

A framework consisting of two themes (background and operational requirements) was finally extracted. The “Background” theme encompasses the basic and infrastructural concepts with three subthemes of 1-laws and regulations 2- NGOs and 3- socio-cultural factors. The second theme was included practical and operational concepts such as preparedness, response, retention, termination and follow-up (Table 1).

**Table 1:** The final list of themes and codes related to managing Iranian healthcare volunteers in disaster

Themes	Subthemes	Codes
1. Background	1–1 Laws and regulations	Laws
		Safety standards and regulations
		Insurance
		Code of ethics
	1–2 NGOs	Creation of NGOs
		Reforming the structure of NGOs
		NGOs–government relations
	1–3 Socio-cultural factors	Loyalty of volunteers
		Community’s viewpoint on volunteers
		Manager’s viewpoint on volunteers
2. Operational	2–1 Preparedness	
		Promote volunteering
		Recruit
		Creating DMATs(Organizing)^a^
		Empowerment
	2-2 Response	Rapid assessment
		Recall and dispatch
		Division of labor
		Coordination
		Communication
		Commanding
		Controlling
		Evaluation
		Feedback
	2–3 Retention	Motivating
		Safety and security
	2–4 Termination	Leaving and dismissal
		Discharging
	2–5 Follow -up	Physical health status
		Mental health status

^a^Disaster Medical Assistance Team

### Theme 1: background

#### Laws and regulations

Majority of experts mentioned that laws and regulations were effective and predisposing factors in the management of volunteers in the healthcare system of Iran during disasters. Moreover, supportive laws and regulations considerably reduce the problems and challenges of the management. The interviewees also strongly emphasized the need to resolve the legal gaps of the recruitment of healthcare system volunteers, particularly in disasters. They also believed that developing safety standards and regulations, arranging insurance and setting codes of the ethics related to the use of healthcare volunteers was among the first measures to be taken in the management of health care volunteers.

*"The first thing to do is to have clear volunteer recruitment laws and regulations dealing with the recruitment to follow up of the volunteers. It must be systematic, and the recruitment, insurance, and follow up of the volunteers should be based on a legal provision or regulations or instruction issued. "(Medical department in Iranian Red Crescent)*

#### Non-governmental organizations (NGO)

Regarding this dimension, interviewees referred to strengthening the role of NGOs in disaster risk reduction by reforming the structure of the NGO’s to be more agile and reinforcing mutual trust and coordination between the NGO’s and governmental agencies, which are responsible in managing disasters.

*"In Iran, NGOs are not given much importance and they do not play a decisive role in disaster management; nevertheless, it is the duty of NGOs to identify, recruit and organize people in developed countries" (An NGO member).*

#### Socio-cultural factors

Some participants pointed to a decline in the volunteering motivation at the societal level as well as decreasing the total number of volunteers, so this issue can affect the number of professional and expert volunteers:

*"Reduced volunteering motivation has also become a challenge in recent years, particularly in cases of human-made incidents, I think; we have to increase volunteering motivation through different training methods and plans" (A researcher in volunteerism)*

Reducing trust in the performance of volunteers among managers was among other issues referred to by the interviewees that lead to decrease mutual trust between managers and volunteers:

*"The culture of using volunteers among managers and the injured people's attitude toward volunteers and volunteering can be better, and if we want to enhance the volunteers’ participation, we should consider these issues that are somehow considered as a socio-cultural factor (Hospital manager)"*

### Theme 2: operational requirements

#### Preparedness

Considering the importance of quick response to the injured, the vast majority of interviewees believed that plans should have been designed in the preparedness phase for timely and effective use of volunteers; therefore, it is possible to employ pre-identified volunteers and organize them as professional teams like DMATs (Disaster Medical Assistance Teams).

Moreover, based on the interviews, the plans in the preparedness should encompass some actions for promoting volunteering and encouraging the people to be volunteer, recruiting, organizing and empowering them.

*"It is better that the Ministry of Health, as a trustee of healthcare volunteers, design a comprehensive plan to register and prepare volunteers, and also make the volunteers ready during the preparation phase so that they can be activated and dispatched when needed according to that plan" (Iranian Red Crescent member).*

#### Response

According to the interviewees, in the response phase, it is better to make a rapid assessment of the affected area before any deployment. Clear procedure of recalling and dispatching volunteers as well as the necessity of division of labor between healthcare volunteers and clarified job description were also considered as the necessary measures at this stage. Coordination, communication, commanding, controlling and evaluating the volunteers (daily and at the end of the mission) and providing feedback were another part of the plan proposed by the interviewees.

*"... in this plan, cases such as how the needs assessment should be carried out, how to recall and organize them, and how to write their tasks must be pointed out" (Iranian National Emergency Organization)*

#### Retention

According to the interviewees, the volunteers’ retention rate was based on whether or not there was a plan for motivating the volunteers. It also depends on if the safety and security concerns of volunteers are addressed. In this regard, it was suggested that, in addition to providing training programs for the entire health system community, the volunteer dignity should be respected at all stages:

*"I saw an interesting mechanism in Sudan, where volunteers worked for a humanitarian organization; however, they went to Kenya to have fun after three weeks of hard work in an insecure area, we can also design such incentives for them to have highly motivated volunteers (An NGO member)."*

#### Terminating

According to the interviewees, it is better to have a plan to terminate the volunteering period and in this plan, issues ranging from volunteers’ discharge terms and release of volunteers who do not have acceptable qualification to work:

*"The terms based on which the commander can expel the volunteer should be specified in an agreement signed with the volunteer before the recruitment phase (Emergency Medical Center)."*

Nevertheless, it is necessary to anticipate procedures to substitute volunteers who expelled or left the mission:

*"If part of the workforce cannot carry on the mission for any reason and even if the volunteers get tired after a while and then leave the mission, we should have Plan B and immediately call the alternative forces, for whom we have already planned for" (Researcher in volunteerism)*

#### Follow-up

Upon completion of the mission, the issue of following up the volunteers who participate in the mission should be given first priority in the health care volunteer management plan, their potential problems should be addressed and their physical and mental health status should be assessed during and after completion of the mission:

*"Volunteers should not be left on their own after the mission. I think that the healthcare volunteers need to be followed up more than other volunteers since they are more sensitive in this issue and they are exposed to physical and mental harm more than the other groups" (An NGO member).*

Follow-up should be carried out regardless the fact that the volunteer has abandoned the field without prior notice or has been disqualified or discharged:

*"Some people may believe that it is not necessary to follow up volunteers who are absent after two or three days of work; however, I believe these volunteers should be a priority, because they might be absent due to mental or physical problems” (A manager in Tehran Disaster Mitigation and Management).*

### Delphi results

In this phase, 42 experts were involved in the survey (age 45.8 ± 8.6). According to the results of the first round, the importance of all items was above 75% (Table 2) consequently, there was no need to run the second round of the Delphi and all items were included in the third round.

**Table 2:** The importance of items related to managing healthcare volunteers in disasters

Items		Importance (First round) *N* = 42%	Consensus rate (Second round) *N* = 38%
Laws and regulations	Passing the related law	81	100
	Comprehensive safety standards and regulations	89	100
	Insurance coverage for volunteers	89	97.5
	Developing code of ethics	80	87.5
NGOs	Facilitate creation of NGOs	81.4	100
	Reforming the structure of NGOs	78.6	77.5
	Strengthening the NGOs–government relationship	84.2	97.5
Socio-cultural settings	Enhancing loyalty of volunteers	83.8	100
	Working on community’s viewpoint on volunteers	85.8	100
	Working on manager’s viewpoint on volunteers	89.6	97.5
Preparedness	Promote volunteering	84.2	100
	Set recruiting guideline	92.8	85
	Organizing DMATs	87.6	95
	Empowering the volunteers	93.8	100
Response	Conducting rapid assessment	90.4	100
	Recall and dispatch	92	100
	Division of labor	93.8	100
	Defining job description	92	97.5
	Coordination between volunteers and formal responders and commander	89	98
	Anticipating communication methods	95.2	98
	Commanding	90	96
	Controlling volunteers	84	97.5
	Evaluating volunteers	80	94
	Providing daily feedback to volunteers	75	70
	Providing feedback to volunteers at the end of mission	80	100
Retention	Motivating volunteers	82.4	100
	Providing safety and security	93	100
Termination	Anticipating leaving and dismissal guideline	85.8	100
	Anticipating discharging guideline	80	90
Follow -up	Physical health status	88	100
	Mental health status	89	100

In the final round, the experts were asked for their final opinion about items. All of the items in this round had enough consensus rates and were confirmed.

**Fig. 1:**
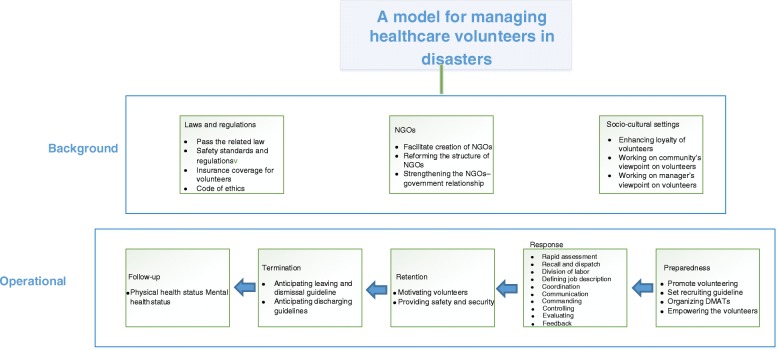
The proposed model of managing Iranian healthcare volunteers in disaster

After identifying the conceptual components of the model in two Delphi rounds and then achieving the acceptable consensus rate, the following model was developed (Fig. 1).

## Discussion

According to the results of this study, to manage healthcare volunteers at the time of disasters, there should be some background and operational requirements. In the background theme, the dimensions were supportive laws and regulations, paying more attention to sociocultural factors and the use of NGO capacity as the contributor in management of volunteers. Moreover, in the second theme the emphasis was on having the preparation, response, retention, termination and following up plans for managing health care volunteers.

The results of a study conducted in Iran revealed that there was no mutual trust between the NGOs and governmental agencies and perhaps this issue has led to the less highlighted role of the NGOs in disasters in Iran. Nevertheless, other studies had also referred to some pitfalls occurring during the recruitment of the NGOs in disasters, such as unspecified and inefficient structures of Iranian NGOs [28, 29]. Regarding public view toward volunteers, Hennesy pointed out the need to train people to foster the culture of collaboration and Bjerneld et al. mentioned the need to train volunteers to have more self-confident volunteers. In Iran, there is no legislation related to disaster-volunteering services. Existing legislations on disaster management also respond to the traditional measures and they still did not enter the dimensions of readiness and risk reduction. Even in the Iranian disaster management organization law adopted by the Islamic Consultative Assembly in 2008, as well as its amendment of 2017, the role of volunteers’ participation in accidents has not been mentioned [30].

Regarding culture and public training in order to increase the quality and quantity of volunteers, an inter-organizational coordination is required [31]. On the other hand, lack of coordination between the sectors involving in disaster management in Iran [32], it seems that a comprehensive plan with the participation of relevant organizations such as the Ministry of Health, The Red Crescent and the National Disaster Management Organization is needed.

Health volunteers, like other volunteers, significantly must be considered [31]. Additionally some studies referred to giving motivation to volunteers [33, 34] and ensuring their safety as effective strategies to enhance their retention rate in the mission [31, 35]. Considering safety seems to be more important for health volunteers compared to other volunteers [12]. Therefore, it seems that motivating the volunteers and retaining their dignity and ensuring their health and security should be included in the plan.

According to the results, terminating the volunteers should be integrated in the healthcare volunteer management plan, though it has been less addressed in previous studies. Furthermore, some studies as well as this study pointed out the necessity to follow up post-mission physical and mental health status of the volunteers [36–38].

Overall compared with other related models in managing volunteers [18, 21], in our model there were some aspects including following up the health status of volunteers, giving feedback and providing safety and security of the volunteers that have not been reported in the previous studies.

The main limitation of this study was the impossibility of conducting interviews immediately after the disasters; therefore, to overcome this limitation, the researchers interviewed the experts who experienced the disasters. The paucity of studies in the health care volunteers in the world and lack of high quality literature in this field in Iran were among the other limitations of the study. This limitation forced the authors to consider all aspect of the health care volunteers in the study.

## Conclusion

The aim of this study was to develop appropriate conceptual model for managing healthcare volunteers and the results show that for designing the volunteer management plan, there are some requirements such as providing supportive laws and regulations, closer collaboration between governmental organizations and NGOs, fostering culture of volunteerism, as well as having plans at the operational levels.

Consequently, the healthcare volunteer management plan seems to be initially supported by comprehensive law and credible regulations. This could be done with the participation of the Ministry of Health and the National Disaster Management Organization of Iran and the Islamic Consultative Assembly.

In addition, the NGOs role as an interface between health care volunteers and governmental agencies should be strengthened. In this regard, it is recommended to form Social unit in the Ministry of Health, this unit is expected to play an effective role in reducing the gap between The NGOs and Ministry of Health as a leading agency in delivery of health care in disasters.

The main recommendation of this study is that volunteer preparedness plans should be integrated into the risk reduction programs of the health system, the capacities created to use health system volunteers should be employed during the response phase, and following up the volunteers’ physical and mental health status after the mission should be prioritized in post-response phase measures. It is suggested that all of these items should be taken into account in the general policies of the Iranian health system and even in the accreditation of health centers.
