# Correction to: Conceptual model of managing health care volunteers in disasters: a mixed method study

**DOI:** 10.1186/s12913-019-4165-3

**Published:** 2019-05-24

**Authors:** Ibrahim Salmani, Hesam Seyedin, Ali Ardalan, Tahmineh Farajkhoda

**Affiliations:** 1 Department of Disasters and Emergency Health, Research Center of Accidents Prevention and Dealing with Disasters, Shahid Sadoughi University of Medical Sciences, Yazd, Iran; 2 Department of Health in Emergencies and Disasters, School of Health Management and Information Sciences, Iran University of Medical Sciences, Tehran, Iran; 3 Department of Disaster Public Health, School of Public Health, Tehran University of Medical Sciences, Tehran, Iran; 4 Harvard Humanitarian Initiative, Harvard University, Cambridge, USA; 5 Reproductive Health, Research Center for Nursing and Midwifery Care, Shahid Sadoughi University of Medical Sciences, Yazd, Iran

**Correction to: BMC Health Serv Res**

**https://doi.org/10.1186/s12913-019-4073-6**

In the original publication of this article [1], the percent sign at the first row of the Table 2 needs to be deleted. The updated Table 2 is shown below:

**Table 2:** The importance of items related to managing healthcare volunteers in disasters

Items		Importance (First round) *N* = 42	Consensus rate (Second round) *N* = 38
Laws and regulations	Passing the related law	81	100
	Comprehensive safety standards and regulations	89	100
	Insurance coverage for volunteers	89	97.5
	Developing code of ethics	80	87.5
NGOs	Facilitate creation of NGOs	81.4	100
	Reforming the structure of NGOs	78.6	77.5
	Strengthening the NGOs–government relationship	84.2	97.5
Socio-cultural settings	Enhancing loyalty of volunteers	83.8	100
	Working on community’s viewpoint on volunteers	85.8	100
	Working on manager’s viewpoint on volunteers	89.6	97.5
Preparedness	Promote volunteering	84.2	100
	Set recruiting guideline	92.8	85
	Organizing DMATs	87.6	95
	Empowering the volunteers	93.8	100
Response	Conducting rapid assessment	90.4	100
	Recall and dispatch	92	100
	Division of labor	93.8	100
	Defining job description	92	97.5
	Coordination between volunteers and formal responders and commander	89	98
	Anticipating communication methods	95.2	98
	Commanding	90	96
	Controlling volunteers	84	97.5
	Evaluating volunteers	80	94
	Providing daily feedback to volunteers	75	70
	Providing feedback to volunteers at the end of mission	80	100
Retention	Motivating volunteers	82.4	100
	Providing safety and security	93	100
Termination	Anticipating leaving and dismissal guideline	85.8	100
	Anticipating discharging guideline	80	90
Follow -up	Physical health status	88	100
	Mental health status	89	100
